# Application of Convergent Science and Technology toward Ocular Disease Treatment

**DOI:** 10.3390/ph16030445

**Published:** 2023-03-16

**Authors:** Ayça Bal-Öztürk, Ece Özcan-Bülbül, Hazal Ezgi Gültekin, Berivan Cecen, Ebru Demir, Atefeh Zarepour, Sibel Cetinel, Ali Zarrabi

**Affiliations:** 1 Department of Stem Cell and Tissue Engineering, Institute of Health Sciences, Istinye University, Istanbul 34396, Türkiye; 2 Department of Analytical Chemistry, Faculty of Pharmacy, Istinye University, Istanbul 34396, Türkiye; 3 Department of Pharmaceutical Technology, Faculty of Pharmacy, Istinye University, Istanbul 34396, Türkiye; 4 Department of Pharmaceutical Technology, Faculty of Pharmacy, Izmir Katip Celebi University, Izmir 35620, Türkiye; 5 Department of Mechanical Engineering, Rowan University, Glassboro, NJ 08028, USA; 6 Department of Biomedical Engineering, Rowan University, Glassboro, NJ 08028, USA; 7 Nanotechnology Research and Application Center (SUNUM), Sabanci University, Istanbul 34956, Türkiye; 8 Molecular Biology, Genetics and Bioengineering Program, Faculty of Engineering and Natural Sciences, Sabanci University, Istanbul 34956, Türkiye; 9 Department of Biomedical Engineering, Faculty of Engineering and Natural Sciences, Istinye University, Istanbul 34396, Türkiye

**Keywords:** ocular disease, smart contact lens, nanotechnology, drug delivery, convergent science

## Abstract

Eyes are one of the main critical organs of the body that provide our brain with the most information about the surrounding environment. Disturbance in the activity of this informational organ, resulting from different ocular diseases, could affect the quality of life, so finding appropriate methods for treating ocular disease has attracted lots of attention. This is especially due to the ineffectiveness of the conventional therapeutic method to deliver drugs into the interior parts of the eye, and the also presence of barriers such as tear film, blood-ocular, and blood-retina barriers. Recently, some novel techniques, such as different types of contact lenses, micro and nanoneedles and in situ gels, have been introduced which can overcome the previously mentioned barriers. These novel techniques could enhance the bioavailability of therapeutic components inside the eyes, deliver them to the posterior side of the eyes, release them in a controlled manner, and reduce the side effects of previous methods (such as eye drops). Accordingly, this review paper aims to summarize some of the evidence on the effectiveness of these new techniques for treating ocular disease, their preclinical and clinical progression, current limitations, and future perspectives.

## 1. Introduction

The eye is one of the most complex sensory organs in the body and is divided into two main parts, namely, the anterior and posterior segments [1]. The anterior segment is the front one-third of the eye which is composed of cornea, trabecular meshwork, conjunctiva, pupil, iris, ciliary body, aqueous humor, and lens, and the posterior segment is the back two-thirds of the eye that comprises the choroid, sclera, retina, macula, vitreous humor, and optic nerve [2]. This complex organ contains several layers of distinct tissues that are prone to a variety of disorders, each of which should be specifically targeted for proper treatment.

**Figure 1:**
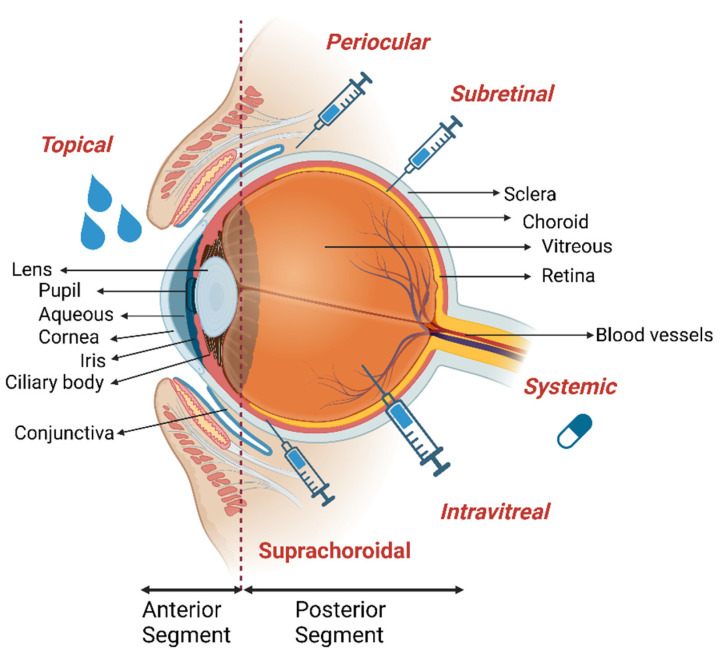
Anatomy of the eye. Anterior and posterior of the eye is given with the major corresponding tissues labeled. Main drug administration routes to the eye including topical, suprachoroidal, intravitreal, subretinal, periocular and systemic delivery are shown. Illustrations were created by using Biorender with agreement #GX24VQ4UJ2.2.

The field of ophthalmology as an advanced discipline provides substantial knowledge about ocular disease mechanisms and treatment strategies. However, the human eye with its complex anatomy and physiological barriers limits the treatment outcomes, especially of topical administrations, resulting in low efficacy [3]. Among the routes of drug administration (Figure 1), topical delivery is the most common and convenient method for ocular diseases due to patient compliance [4]. Here, the drug is directly delivered to the anterior segment of the eye as much as eye blinking, tear film turnover, corneal barrier, and lachrymal secretions allow [3]. Ultimately, less than 5% of the drug can pass through cornea and reach the eye, which almost eliminates this route for posterior eye disease treatment [5]. Additionally, blood-aqueous, blood-retinal barriers, retinal inner limiting membrane, and sclero-corneal parenchyma further limit permeability of pharmacological agents into the eye [3,5]. Alternatively, systemic administrations including intravenous injection and oral dosing are applied to reach the posterior side of the eye, where blood-aqueous and blood-retinal barriers decrease efficiency. Therefore, to reach necessary concentrations of drug in the target area, large doses are required [5]. The limitations of common strategies have led researchers to develop novel targeted drug delivery methods using nanobiotechnology approaches. Nanosized carriers including liposomes, nanomicelles, nanoparticles, dendrimers, nanofibers, viral vectors, nanoemulsions, nanobubbles, and implants have been developed to improve patient compliance, treatment performance, and cost-efficiency [4]. The combined application of this science with technologies such as contact lenses, microneedles, etc., has led to the creation of innovative technologies for treatment of ocular disease with the lowest probable dissatisfaction and the highest performance. According to the above-mentioned features, this review provides an overview of nanotechnology-based systems for ocular disease treatments and novel technologies for ocular drug delivery methods.

## 2. Ocular Anatomy and Diseases

The cornea (Figure 1), with about 0.5 mm thickness, is the transparent outermost cellular layer of the eye and is composed of five distinct layers containing epithelium, Bowman’s membrane, stroma, Descemet’s membrane, and endothelium. Cornea maintains ca. 70% of the refractive power of the eye and protects the eye via providing a barrier towards physical injury, pathogens, and fluid loss [3]. Diseases affecting the cornea may originate from traumatic injuries, infections, corneal dystrophies, and age-related degeneration. Corneal scars, opacities, and haze in this tissue can lead to visual loss [6]. Keratoconus is a common ectatic corneal dystrophy characterized by thinning of corneal stroma and steepening of cornea leading to visual distortion and irregular astigmatism [7,8]. Corneal infections are caused by a variety of microorganisms including bacteria, yeast, protozoa, and viruses. Infectious keratitis can result in opacity due to infection, inflammation, degeneration, nutritional deficiency, and trauma; however, the most common infectious keratitis is developed due to bacterial infections such as *Staphylococcus aureus (S. aureus)*, *Pseudomonas aeruginosa (P. aeruginosa)*, and *Streptococci* spp. [9].

The outer surface of the cornea is covered with a tear film composed of three distinct layers of lipid, aqueous, and mucin [3]. Tear film has a dynamic cycle with high tear turnover (0.5–2.2 L/min) [10]. This lubricant film provides smoothness to the ocular surface for light refraction, protects it against irritants, and supplies oxygen and nutrients for the avascular corneal epithelium. Dysfunction of the tear film can result in dry eye syndrome causing glare, blurred vision, irritation, and hyperemia. In advanced cases, vision impairment is prominent in consequence of inflammation, epitheliopathy, and corneal neurosensory abnormalities [11].

The iris, located behind the cornea and in front of the lens, controls the pupil, through which light passes. Muscles of the iris adjust pupil dilation size according to incoming light intensity [1]. Anatomical abnormalities of the iris such as attachment of iris to the cornea and change in the angle between the iris and the cornea may lead to closed-angle and open-angle glaucoma, respectively [12]. Iris atrophy is an infrequent disease characterized by degenerated areas on the iris. Additionally, inflammations in the anterior chamber and on the iris (iritis also called Anterior uveitis), result in eye redness and eventually visual impairment [13].

The ciliary body, the circular structure behind the iris, is responsible for focusing the lens by the help of the ciliary muscle and producing aqueous humor through secretion from ciliary epithelium. The ocular lens has the ability to focus incoming light due to its elastic and clear structure. Decrease or loss of elasticity, focusing ability, and transparency of the lens result in cataract formation and vision impairment [1]. A cataract is the major cause of blindness worldwide and cataract surgery, based on replacement of the lens with an artificial one, is the only treatment option [14].

The human sclera comprises 95% of the eye’s surface area neighboring the cornea that maintains intraocular pressure (IOP) and globe shape of the eye [2]. Choroid is a dynamic barrier, including vascular and innervated structures on the posterior part of the eye, which lies between retina and sclera. Choroid helps in regulating intraocular pressure, aqueous humor turnover, and thermoregulation. The pigmented choroid layer absorbs light through melanin pigments to decrease light reflection within the eye and light intensity reaching the retina [1]. It can be affected by neovascularization signalized by abnormal growth of blood vessels into the retina [15].

The conjunctiva is a thin membrane that covers the sclera and is found between the eyelid and the sclera. It protects the eye from microorganisms and produces mucus to lubricate and support tear film stability [1]. The conjunctiva can develop allergies to animal dander, airborne pollens, and other environmental antigens. Allergic conjunctivitis is one of the most common eye diseases [16].

The retina is a light-sensitive tissue located at the back of the eye, consisting of glial, neuronal, and photoreceptor cells [1]. Retinal inflammation, angiogenesis, ischemia, and leaky retinal vasculature can be observed in diabetic patients, and these lead to diabetic retinopathy which causes vision loss [17]. Degeneration of the macula, the photoreceptor-rich region of the retina, termed age-related macular degeneration (AMD) leads to irreversible vision loss [1]. Similarly, glaucoma, progressing either with high or low pressure [12], causes loss of retinal ganglion cells and consequent vision loss. The optic nerve maintains communication between the eye and the brain via the extension of ganglion cell axons. The optic nerve transports visual impulses from the retina to the next nerve cell as electrical impulses [1]. Neurological disorders affecting the optic nerve such as multiple sclerosis (MS) can cause vision loss [18].

Normally there are two different ways for drug delivery to the eyes; local injection, which is traumatic and less acceptable and only applied by the medical personnel, and topical administration, which includes different types of eye drops and gels and is more user friendly [19,20]. Despite the efforts made, still there are some limitations in producing high performance topical therapeutic compounds for ocular disease treatment. Indeed, most of the therapeutics are accompanied by eye irritation and redness and visual impairment that annoy patients. Moreover, presence of the tears barrier restricts the bioavailability and penetration of therapeutic compounds on the surface and into the inner parts of the eyes. Repeated administration of the therapeutic agents could also induce other types of disease such as subconjunctival hemorrhage, retinal toxicity and detachment, endophthalmitis, and temporary IOP elevation [21,22]. Thus, it is critical to introduce novel technologies for the treatment of ocular diseases in both anterior and posterior segments. One of the interesting strategies that have been used in recent years for overcoming these limitations is the application of nanotechnology and its combination with other techniques that could lead to an enhancement in the performance of treatment and reduce the probable side effects.

## 3. Topical Nano-Formulations for Ocular Drug Delivery

During the past decades, nanotechnology-based applications have provided a new perspective on treatment of ocular diseases via increasing drug bioavailability, providing controllable release of therapeutic compounds, decreasing eye irritation, and enhancing the performance of treatment by providing up to 100% drug permeability to both anterior and posterior segments of the eye [23]. Several nanotechnology-based treatment options have been developed for ocular diseases which can be counted as nanoparticles, micelles, nanoemulsions, nanofibers, nanobubbles, liposomes, dendrimers, nano-capsules, etc. These nano-formulations could deliver small molecule drugs and biological active molecules such as proteins, antibodies, glycoproteins, and nucleic acids.

The same as other materials, nanomaterials could also induce toxicity effects on eyes; however, their toxicity features are different from bigger compounds that could induce chemical or mechanical injuries. Indeed, their small size could enhance their ocular residence time, due to their easier contact with surface of eyes, and increase their penetration through the cornea (depending on the size of nanoparticles). Thus, clearance of smaller size nanomaterials from the ocular surface is harder than bigger ones. They are more tolerated by the eyes and could penetrate into deeper parts of the eye, pass through the barriers, and induce toxicity and inflammatory effects. Beside size and exposure time, other features of nanoparticles such as their chemical compounds, surface charge, shape, dosage, and their behavior in the biological system could also affect their toxicity, and so it is critical to consider all of these features during the fabrication of nanomaterials for ophthalmic applications [24].

Application of these therapeutic compounds provides the capability of treating diseases related to both anterior and posterior parts of the eyes, as described in detail below.

### 3.1. Treatment of Disease Related to the Anterior Segment of the Eye

Synthetic polymers such as poly (lactide-co-glycolide) (PLGA), poly (caprolactone) (PCL) and poly (lactic-acid) (PLA), natural polymers such as dextran, chitosan, and hyaluronic acid, and their combination form are commonly used for nanoparticle-based delivery systems to deliver protein, glycoprotein, and peptide-based therapeutics [25,26]. For instance, lactoferrin, an abundant anti-inflammatory protein of ocular tissues, was incorporated into PLGA nanoparticles to increase its stability in the aqueous environment of the eye. One third higher corneal permeability and improved release duration and anti-inflammatory activity were confirmed by both in vitro and in vivo tests [27]. Similar to synthetic polymers, natural polymers were used, alone or in combination with other polymer or lipid molecules, to obtain the necessary characteristics for drug delivery. In a topical administration formulation, betamethasone sodium phosphate, an anti-inflammatory drug, was loaded into the alginate nanoparticles. Then, to control the drug release efficiency and improve the bioavailability of the nanoparticle inside the eyes, two different polymeric coatings were applied: gelatin and chitosan. While both nanoparticles exhibited adequate encapsulation efficiency, the gelatin-coated ones had the highest mucoadhesive strength of about 75% [28]. It was revealed in this study that the adhesion feature of the nanoparticles resulted from both the electrostatic interactions and the functional groups of the surface charge of the nanoparticles and mucins.

Nucleic acid-based delivery is used in another novel type of nanoparticle, which has been utilized in topical administration for the treatment of ophthalmic diseases. The treatment was applied in a micelle form nanostructure, in combination with a lipid core for the delivery of neomycin B and kanamycin B. DNA nanoparticles showed increased adherence of both neomycin B and kanamycin B to cornea with no toxicity and 25 times lower concentration of drug in comparison to the clinically used drops [29].

Nanomicelles have an amphiphilic structure comprising a hydrophobic core and hydrophilic shell and they have the ability to encapsulate drugs for ocular delivery [30]. For instance, a combination of water-soluble chitosan and hydrophobic palmitic acid were used in a study to fabricate nanomicelles. Modification of chitosan/ palmitic acid nanomicelles with N-acetylcysteine enhanced their mucoadhesion ability for topical administration. The presence of positively charged chitosan from one side, and the thiolated polymer from the other side improved the bioavailability and adhesion ability of this nanomaterial. Encapsulation of the anti-inflammatory non-steroidal drug, flurbiprofen, inside these particles showed 2.44 fold higher permeation by adhering to the mucin layer ex vivo and 95–80% retention rate on the cell surface of monolayer and three-dimensional spheroids of human immortalized corneal epithelial cells [31]. A synthetic polymer based nanomicelle was also generated for topical ocular delivery. Tacrolimus, an immunosuppressant, loaded amino-terminated poly (ethylene glycol-block-poly(D, L)-lactic acid) (NH2-PEG-b-PLA)/ HPMC nanomicelles exhibit efficient in vitro permeation (approx. 2 fold higher permeation than tacrolimus suspension drop) and in vivo retention and inhibition of graft-rejection [32]. In another study, Rebaudioside A (Reb A) nanomicelles were used to enhance the solubility of Pterostilbene (Pt), a natural stilbenoid that protects cells from inflammation and oxidative stress. Pt-loaded nanomicelles provided 6.39-fold greater in vivo intraocular permeability than free Pt and a greater anti-inflammatory effect in rabbit eye and treatment efficiency on corneal alkali burns in mice compared to free Pt [33].

Liposomes are bilayer lipid structures composed of phospholipids and cholesterol which could encapsulate both hydrophobic and hydrophilic therapeutic compounds. Rebamipide, is a hydrophilic drug used for the treatment of dry eye disease. It is used in the form of eye drops; however, the bioavailability of this drug is low, and it is removed from the eye via the nasolacrimal duct. In addition, it could lead to blurry vision due to its opaque and turbid appearance and the higher doses of this drug also caused irritation. Utilizing eye drops containing Rebamipide loaded liposomes showed better stability, controlled release rate, and better tolerance by the patients. Application via liposomes could improve the bioavailability of the drug in the cornea, enhance its effectiveness, and reduce the dosage and irritation effects [34].

Nanofibers are another nanotechnology to improve conventional drug treatments by increasing retention time. Additionally, nanofibers consist of an extensive interconnected fiber network and the pores allow incorporation of multiple drug molecules and thereby eliminate drug resistance problems and multiple drug applications. Fluconazole and Cinnamaldehyde loaded two-layer polyvinyl alcohol and gelatin electrospun nanofibers exhibit antifungal and antibiofilm activities simultaneously inhibiting biofilm formation of Candida albicans up to 49% [35].

Incorporation of nanoparticles into the structure of nanofiber nets is also possible. For instance, nanoparticle-modified nanofibrous wound dressing membranes offer sustained drug release without scar formation on the cornea. PLA electrospun nanofiber membrane modified with silver nanoparticles and cellulose nanofibrils exhibited antimicrobial activity and enhanced cell proliferation. The presence of silver nanoparticles provided growth inhibition of *Escherichia coli (E. coli), S. aureus*, and *Fusarium* spp. of over 95%, 95% and approx. 75%, respectively. Additionally, co-cultured conjunctival epithelial (CjECs) and corneal epithelial cells showed higher viability on silver nanoparticle-incorporating PLA nanofibrous membranes compared to PLA nanofibrous membranes. Owing to the biocompatibility, bactericidal and antifungal effects of silver nanoparticle incorporating PLA electrospun membranes, they are considered a promising ocular wound healing bandage for corneal and conjunctival wounds [36].

### 3.2. Treatment of Posterior Segment Eye Disease

Mucoadhesiveness of a drug is important for its topical administration to address posterior segment diseases in order to reduce frequency of drug administration. PLGA-PEG nanoparticles coated with a cationic lipid shell, with features such as enhanced mucoadhesion, prolonged corneal retention, and improved bioavailability of the loaded drug, melatonin, were used in another study that showed no sign of ocular irradiation or cytotoxicity in vivo [37]. In other words, the interaction between positively charged lipid and negatively charged mucin improved the interaction of this system with the cornea.

The permeability of the drug is one of the significant properties for the topical administration of drug delivery applications. In another study, chitosan and hyaluronic acid nanoparticles were used to encapsulate erythropoietin (EPO) for its topical administration as an alternative to conventional systemic, intravitreal, and retrobulbar routes. EPO-encapsulated nanoparticles showed 23.7–60%, 22.6–85.3% and 2.5 times higher permeated EPO amount in conjunctiva, sclera and cornea compared to previous studies, respectively, ex vivo [38].

Nanoparticle penetration efficiencies through ocular barriers and targeted deliveries can be improved by additional agents for topical administration to address posterior eye diseases. For instance, a nonxanthine adenosine receptor antagonist of ciliary body, ZM-241 385, was used with chitosan to coat hollow ceria nanoparticles. Hollow ceria nanoparticles provided sustained release of pilocarpine, whereas dual coating lead to ~250-fold greater bioavailability due to enhanced corneal penetration and targeted delivery to the ciliary body [39].

Lipid-modified DNA micellar nanoparticles can also be used for posterior drug delivery applications through topical administration. The efficiency of lipid-DNA nanoparticle micelles was examined using anti-glaucoma drug travoprost for glaucoma treatment and fluorophore-DNA conjugates for fluorescent imaging. The non-covalent binding between DNA nanoparticles and travoprost increased residence time resulting in four times higher accumulation compared to the free drug four hours after application on the cornea of ex vivo pig and in vivo rat models. These results showed that DNA-based nanocarriers are promising candidates for drug delivery systems in ocular diseases [40].

Theranostic systems provide diagnosis and treatment simultaneously and have been applied for ophthalmological applications. In the case of posterior segment ocular diseases, they could overcome the difficulties in treatment and diagnosis of angiogenesis-related disorders. Bi (bismuth) and SiO_2_ (silicon oxide) coated upconversion nanoparticles were encapsulated into penetratin (PNT) peptide and hyaluronic acid (HA) functionalized liposomes. Genistein (GE), an anti-angiogenic agent, was loaded into the liposomes for the treatment of angiogenesis-related diseases. The targeting detection ability and efficiency of the drug delivery system were confirmed in human retinal pigment epithelial cells. In addition, the effectiveness of this new theranostic compound in detection of angiogenesis-related posterior segment diseases was confirmed [41].

Nanoemulsions are composed of oil and aqueous phases, with addition of surfactants to decrease interfacial tension between phases. Ocular drug delivery with nanoemulsions may confer prolonged release and high ocular permeability with the potential to decrease dosage. Travoprost-loaded nanoemulsion composed of Labrafac™ lipophile oil, Tween 80, and water, was shown to extend intraocular pressure reduction interval with ~2-fold higher corneal absorption compared to conventional eye drop Travatan^®^ for treatment of glaucoma in a rabbit model [42].

A nanoemulsion formulation was suggested for age-related macular degeneration (AMD) treatment by ocular injection delivery of anti-inflammatory and antiangiogenic drug triamcinolone acetonide (TA). Loading TA into cationic nanoemulsion increased corneal penetration and bioavailability of TA with about 60% release of initial TA in 180 min following administration. Oil-in-water nanoemulsion composed of soybean oil, Tween 80, DABCO, glycerol, poloxamer 188, and water showed no neovascularization and no irritation 24 h after administration on chicken membranes and the rabbit model [43]. Further studies showed that the nanoemulsion resulted in an approximately 1.5-fold decreased vascular endothelial growth factor (VEGF) secretion and tumor necrosis factor alpha (TNF-α) cytokine production compared to control cells in an AMD model [44]. Another oil-in-water nanoemulsion was generated with almond oil, Tween 80 or Tween 20 as non-ionic surfactants, and Palitzsch buffer for topical administration. Nanoemulsion constituted with Palitzsch buffer exhibit non-irritant properties compared to nanoemulsion constituted with HEPES buffer. On the other hand, Tween 20 enhanced mucoadhesive properties and diffusion into the mucus network of the eye compared to Tween 80 due to differences in charge and size. Both formulations formed stable nanoemulsions and showed no cytotoxic effect against Y-79 (human retinoblastoma cell line) cells [45].

Solid lipid nanoparticles (SLNs) are another class of lipid-based nanoparticles that have the capability of delivering hydrophobic drugs, improving their bioavailability, controlling their targeted release, and enabling the production of drug loaded samples on a large scale [46]. It was used in the formulation of eye drops used for the treatment of AMD, for encapsulating hydrophobic atorvastatin. SLNs could enhance the solubility and stability of the drug inside the eyes along with providing the capability of autoclaving the drug loaded SLNs and enhancing the penetration of samples into deeper parts of the eyes [47]. Positively charged SLNs were also used as a carrier for Natamycin that could improve the penetration of the therapeutic compound into the cornea, prolong the releasing time, reduce the dosage, and have no irritating effects. The lipophilic nature of these nanoparticles along with their small size and positive charge make them appropriate for penetrating through the barriers. They also showed mucoadhesiveness features that could enhance the bioavailability of therapeutic components inside the eye [48].

Combined therapies can enhance retention time of drugs on the corneal surface while increasing drug solubility. For instance, hydrophobic drug solubility can be increased due to the hydrophobic core of nanomicelles, and sustained drug release can be achieved with nanoemulsions due to increased retention time. The combination of nanoemulsions and nanomicelles provides enhanced efficiency compared to their sequestered utilization [49].

Although topical administration is a preferred method for patient compliance, in some applications other routes of administration are inevitable. Intravitreal injection is one such application route that eliminates ocular tissue penetration obstacles in addition to other nanotechnology driven advantages. Drug formulations delivered directly to the vitreous humor might be in various forms including nanoparticles, hydrogels and nanobubbles. In one study, betamethasone phosphate (BetP) and anti-VEGF-loaded injectable antibody-loaded nanofiber hydrogel reduced local inflammation and angiogenesis in wet-AMD cell and animal models [46].

Hydrogel enabled high drug administration dosage and long-term drug release from 15 min to 14 days resulting in reduced application intervals up to single administration. Nanobubbles, on the other hand, have an intrinsic movement capacity in vitreous humor allowing drug molecules to distribute evenly and reach to target sites in the back of the eye. For instance, rhodamine-tagged gas-entrapped nanobubbles were shown to migrate to the posterior segment of the vitreous by multiple corneal ultrasound cycles in both bovine and porcine eyes [47]. Similarly, dextran-based oxygen-containing nanobubbles were suggested as effective treatment for central retinal artery occlusion (CRAO), where the lack of oxygen in the inner retinal layer causes vision loss. Nanobubbles demonstrated high recovery in both retinal precursor cell lines and a hypoxic rat eye model. Their efficiency was also confirmed with oxygen distribution measurements and electroretinography [48].

Nanobubbles can also be used as a mechanical treatment approach and can be formed within the eye. In one study, vapor nanobubbles were generated by gold nanoparticles in the posterior segment of the eye upon pulsed-laser illumination. Generating vapor nanobubbles mechanically ablates opacities in vitreous humor piece by piece. Nanotechnology-based photoablation showed excellent therapeutic effect for vision-degrading myodesopsia that was followed with nanoparticle mobility and photoablation efficiency assays in human vitreous humor, with no aberrant cytotoxicity in Human Müller cells [49]. Antibody-based therapies are applied to prevent or treat ocular angiogenesis prognosis observed both in posterior (diabetic retinopathy (DR), retinopathy of prematurity (ROP), age-related macular degeneration (AMD)) and anterior segment (corneal) neovascularization diseases [50,51]. Vascular endothelial growth factor (VEGF) is an angiogenic stimulator and regulates vasculature homeostasis in ocular tissues. Bevacizumab is a VEGF directed humanized monoclonal antibody that has been used to treat ocular vascular diseases by counteracting VEGF. The route of administration varies depending on the affected ocular tissue. It was shown that bevacizumab-encapsulated PLGA nanoparticles decrease abnormal VEGF-mediated endothelial cell migration, proliferation, and tube formation in vivo. Residency of bevacizumab was prolonged up to 13.53 ± 2.49 days via subconjunctival injection of bevacizumab-encapsulated PLGA nanoparticles into the mouse eye, that indicated enhanced efficiency for addressing both retinal and corneal neovascularization [52].

Despite several studies on the application of different types of nanomaterial for ocular disease, there is no clear evidence about the superiority of one class of nanoparticles against others. For instance, although polymeric nanomaterials are the most diverse and biocompatible class of nanomaterials with different outstanding features, they could affect vision. SLNs show low stability and could be uptaken by the mononuclear phagocytic system, nonspecifically [53]. Thus, more and more studies are needed to find the best classes of nanomaterials for ocular applications.

## 4. Combination Use of Nanotechnology and Other New Techniques for Ocular Drug Delivery

### 4.1. Contact Lenses, Methods of Preparation and Desirable Application

Contact lenses (CLs) are transparent hemispherical devices placed over the cornea and could be classified based on different characteristics, for example, according to their stiffness (soft and rigid CLs), or hydrophilicity (hydrophobic and hydrophilic types) [54]. There are three main methods for the fabrication of CLs including injection molding, lathe-cut, and spin casting methods [55,56]. The lathe-cut technique is the most prevalent method used for all soft and rigid CLs with an anhydrous state, in which high temperature is used for the fabrication of CLs and shaping is done on a lathe after the mass is cut into a cylindrical shape. In the spin casting technique, the mold is filled with liquid monomer at first, and then centrifugal force is used at a controlled rate until the lens takes the desired shape and polymerization is achieved. It is faster and cheaper than the lathe-cut method. The injection molding method is suitable for large-scale production in which liquid monomer is injected between the male and female mold, followed by connecting them, and then lens is cured. As in the spin casting method, the process is conducted under nitrogen gas, so that oxygen degradation does not occur on the surface [54]. The disadvantage of this method is that rapid polymerization creates short chains and reduces efficiency. All of these three methods have salt hydration and polishing as the final standard features [54,55].

In society, CLs are generally used to improve vision, but the use of CLs is not limited to vision or cosmetic purposes. Indeed, CLs are used for the treatment of various complications, such as dry eye disease, age-related macular degeneration, and glaucoma [54]. The lens materials, hydrophobic polymers, repel water that makes up most of the tear surface, which can lead to dryness and cause an albumin film to build up on the lens; this can eventually cause irritation and infection. In addition, the prepared lenses should allow the passage of oxygen to the eye [57]. Oxygen permeability is the property of a lens material that allows penetration of oxygen to reach the eyes. It increases with increasing water content in hydrogel contact lenses, whereas it decreases with increasing water content in silicone contact lenses. The water content of poly(hydroxyethyl methacrylate) (pHEMA) is directly proportional to the decrease in refractive index and oxygen permeability [58]. The oxygen permeability of soft hydrogel lenses is improved by using silicone materials such as polydimethylsiloxane (PDMS) and tris-(trimethyl-silyl-propyl-methacrylate) (TRIS) [59]. With all these developments, the use of drug-loaded contact lenses in ocular controlled drug release and biosensing applications provides overwhelming benefits to overcome the limitations of eye drops, such as rapid release, residence time, and precorneal drug loss [58].

There are different approaches used for the development of drug-loaded CLs; the most well-known are the soaking method, molecular imprinting, Vitamin E-modified CLs, and nanomaterial-incorporated lenses [55,60]. Some of these methods are described in the following sections.

#### Combination Use of Different Types of Nanomaterials Inside Contact Lenses

Nanomaterials are used in ocular drug delivery to ensure that the drug remains in the cornea for a long time, prevent drug breakdown by enzymes, and reduce the drug leakage that may occur during storage and sterilization [54,55]. The drug release occurs via a two-step process; at first, the drug is released through the nanomaterial and reaches the lens matrix, and then it is released from the matrix and reaches the tissues. However, diffusion of drug during storage, reduction in transparency (due to aggregation), and burst releases during use could be considered as disadvantages of this technique [55].

**Figure 2:**
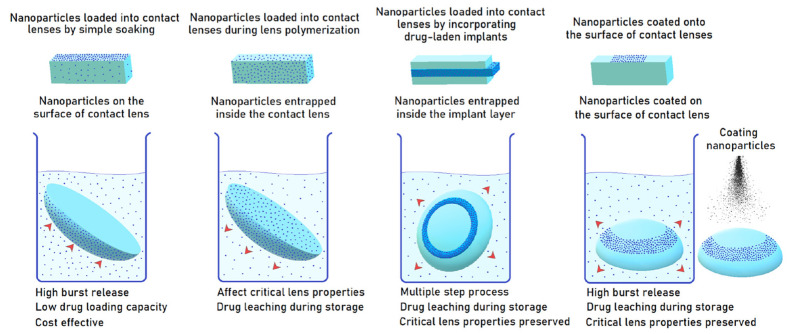
Four methods for manufacturing nanomaterial-loaded CLs. Reprinted with permission from Ref. [61] Advances and challenges in the nanoparticles-laden contact lenses for ocular drug delivery 2023, Elsevier.

Four different methods can be used for the preparation of lens contained drug loaded nanomaterials (Figure 2): (A) Soaking the preformed CLs in the suspensions of nanoparticles, (B) Producing lenses via polymerization of monomers of nanomaterial mixtures, (C) Adding drugs and surfactants to the mixtures to form micelles during the polymerization process, and (D) Immobilizing drug-loaded nanomaterials on the CL surface via chemical bonds. So far, various nanomaterials such as polymeric nanoparticles, liposomes, micelles, and microemulsions have been loaded into the CLs [55]. Some of the ocular studies which used these nanomaterials are given in Table 1.

As mentioned above, one of the main advantages of utilizing nanoparticle-implemented lenses is to prolong drug release in the eye [60]. Keratoconjunctivitis sicca, also known as dry eye, is frequently seen in the community. Mun et al. have developed a cholesterol-hyaluronate micelle-embedded CL. The CL is produced by photopolymerization using ethylene glycol dimethacrylate as a crosslinker. In this way, cyclosporine was released in a controlled manner for more than 12 days. Corneal fluorescent staining, Schirmer tear test, and MMP9 fluorescence analysis were performed to show the effects on dry eye syndrome in diseased rabbits [62]. As is clear, using drug loaded contact lenses can lead to reduced drug administration frequency and so could be more acceptable to patients. One of the other interesting fields in which drug loaded contact lens could be applied is in treating ocular infections.; there are studies in which liposomes and micelles are designed for the treatment of ocular infections. Liposomes are highly biocompatible nanoparticles with the ability to carry hydrophobic drugs in their aqueous core and hydrophobic drugs in the phospholipid shell; however, their phospholipids are prone to oxidative degradation, their stability is lower than polymeric nanomaterials, their volume is limited, and their cost is high [78]. A recent study demonstrated the antibacterial activity of 2-, 5- and 10-layer liposome-loaded CLs containing levofloxacin against *Staphylococcus aureus*. Lenses containing liposome loaded drug showed sustained drug release during 6 days, which is good for ocular drug delivery [79]. Another study with levofloxacin observed that friction caused by eyelid blinking in a drug-loaded PHEMA-based lens did not significantly affect medicine release from liposomes [64]. Hu et al. reported that the drug loading into the micelle increased as the polymer concentration increased, but the transparency decreased [65].

Another material that can be used when preparing CLs to provide controlled ocular drug release is a hydrophobically modified N,N-dimethylacrylamide (DMA) hydrogel. This hydrogel is obtained by free radical copolymerization technique of 2-(N-ethylperfluorooctanesulfonamido) ethyl acrylate (FOSA) and DMA. It is a heat-treatable material as it is a cross-linked hydrogel. In addition, it has been shown that the diffusion of the drug in lenses prepared using DMA/FOSA hydrogel is less sensitive to the pH of the medium [80]. There are also other studies on surface modification. Kazemi Ashtiani used chitosan conjugated poly (2-hydroxyethyl methacrylate) (PHEMA) for CL application. The primary purpose here is to increase the affinity of anionic drugs in eye diseases. Chitosan modification induced continuous ascorbic acid release and increased drug loading efficiency. In addition, surface modification with chitosan has been shown to inhibit protein and bacterial accumulation on the CL [81]. In another study, they modified PHEMA based hydrogels that are widely used in biomedical applications. The aim is to avoid protein components and bacterial accumulation in tears by providing surface modification of the hydrogel with different functional groups, such as carboxylic acid, primary amine, and quaternary ammonium. The data showed that positively charged ammonium and amine groups resist bacterial aggregation and protein adsorption more than alcohol and carboxylic acid groups [80].

Glaucoma, a posterior segment eye disease, is the second leading cause of blindness in the world after cataract. It is treated with eye drops that lower intraocular pressure but this has significant shortcomings such as low patient compliance and low bioavailability. CLs that release glaucoma drugs for a long time have been studied for this purpose. Timolol is a non-selective β-adrenergic blocker used for management of glaucoma. Propoxylated glyceryl triacylate nanoparticles loaded with timolol were dispersed in silicone hydrogel CLs by Jung et al. In vivo studies with beagles have demonstrated the safety and efficacy of long-term use of these lenses [69]. Maulvi et al. loaded timolol onto gold nanoparticles and overcame the disadvantages, such as burst oscillation. The critical features of the lens, such as optical transmittance and swelling, remained unchanged despite the presence of gold nanoparticles [70]. Kumar et al. formulated Eudragit nanoparticles loaded with levobunolol by a soaking method. Loaded lenses’ physical and optical properties were not affected, and controlled long-term release was achieved for levobunolol [71]. In a rabbit tear fluid model, silicone CLs containing brimonidine-loaded nanoparticles were prepared by Xu et al. A high concentration of brimonidine was achieved for 96h with nanoparticle-loaded lenses [72].

Polymeric micelles have a mucoadhesive structure, a hydrophobic core and a hydrophilic shell (10–200 nm) formed by the self-assembly of polymers [82]. One study developed micelle-loaded CLs capable of simultaneously providing sustained release of timolol and latanoprost, and drug release was observed for 144 and 120 h, respectively. Compared with eye drops, the bioavailability of timolol and latanoprost increased by 2-fold and 7-fold, respectively [73]. Microemulsions are thermodynamically stable dispersions of aqueous and oily phases, prepared with co-surfactants and surfactants, with a droplet size of less than 100 nm. They are preferred because of their long duration of action, high ocular absorption, easy preparation, and controlled drug release [55,83]. Wei et al. reported that microemulsion did not change the swelling property and permeability of the lens compared to lenses without microemulsion; they reported that twice as much of the drug could be loaded [74]. In another study by Xu and Liu, drug release was found to last for 36–48 and 48–120 h, respectively, in CLs without microemulsions and lenses containing microemulsions. It was reported that the release was prolonged [75]. In another study, the drug loading capacity was doubled with microemulsions and the drug release time increased approximately 2 times [76].

CLs can also be used for theranostic purposes. A smart CL can be used as an excellent interface between the human body and an electronic device. In one study, Keum et al. developed smart CLs for continuous glucose monitoring and the treatment of diabetic retinopathy. It contains a microcontroller chip and flexible electrical circuits that can provide real-time electrochemical biosensing and controlled drug delivery on demand. This non-invasive method has the potential for the diagnosis of diabetes by conventional invasive blood glucose testing and the treatment of diabetic retinopathy in diabetic rabbit models [84]. In another study, Yang et al. prepared a wireless theranostic CL that can monitor intraocular pressure wirelessly and release drugs when requested. The wireless intraocular pressure sensing module precisely detects intraocular pressure fluctuations thanks to its capacitive sensing circuit design. In addition, the drug delivery module enables the release of an anti-glaucoma drug via iontophoresis [85]. In another study, Kim et al. presented a theranostic smart CL containing an integrated circuit chip for monitoring and controlling intraocular pressure. The gold hollow nanowire-based intraocular pressure sensor showed improved strain sensitivity, biocompatibility, and chemical stability. The system can be used to deliver timolol at any time for intraocular pressure control. The developed theranostic smart CL is a good system that can be used as a futuristic personal health platform for glaucoma and other ocular diseases [86].

### 4.2. Microneedles, Their Different Types, and Their Applications for Ocular Disease

Microneedles (MNs) are devices that are made of polymer or metal and have diameters ranging from a few micrometers to 200 μm [87,88]. Because of their little projections, MNs do not damage the tissue severely. MNs can not only overcome the drawbacks of current standard delivery methods, but they can also pass the ocular barriers and transport their payloads directly to the site of action [88,89]. The micron-sized needles have a simple insertion process to the eye, making them suitable for various applications. The application of MNs on the eye is performed with minimally invasive techniques. To deliver drugs into the anterior and posterior sites of the eye, MNs can be administered via intrascleral and/or intracorneal methods. They are commonly applied by intrascleral insertion. It is possible to insert MNs into the sclera manually [90]. On the other hand, microneedle applicators can be utilized to facilitate the process. MN syringes and pens are some of the examples of applicators. MNs can also be applied via ocular patches to improve the permeation and retention of drugs in the cornea [91,92,93]. These MNs needles are less painful than the standard injectable treatments, and the medication they contain may be designed to be released gradually over a lengthy period [94,95,96]. Therefore, it would not be necessary to apply the drugs repeatedly.

#### 4.2.1. Different Types of Microneedles

MNs come in various forms, each of which has a specific therapeutic use. In the case of ocular administration, there are three types of MNs including solid coated, hollow, and dissolving polymeric MNs [97,98]. Solid-coated MNs are the sort of MNs that are meant to pierce the tissue, at which point the coating will quickly disintegrate [99,100]. After that point, they are eligible for removal. Their lack of a hollow core may distinguish these MNs. The primary purpose of solid MNs is to generate holes inside the sclera or the eye’s cornea that produce a channel at the micron scale, enabling the drug to be delivered efficiently and in a targeted manner. Metals such as stainless steel and silicon probes are examples of materials used during the MN manufacturing process [101,102]. The use of these materials for ocular administration has several drawbacks, including the fact that they are not biodegradable, and the construction procedure is complicated.

Hollow MNs are micron-sized needles that solely contain the formulation on the interior of the needles, to ensure that the drug is effectively delivered deep into the ocular tissue. The medicine delivery via hollow MNs may be accomplished using a variety of formulations, such as nanoparticles and microparticles. Hollow MNs are often fabricated using borosilicate micropipette tubes. However, in certain instances, stainless steel or biodegradable polymers are also acceptable alternatives since borosilicate micropipette tube is not regarded as a suitable candidate for use in clinical applications [100,103]. The micropipette puller method is part of the manufacturing process. This technique involves pulling the fabric up with the assistance of a machine to produce hollow space and lengthening the needles [104,105].

**Figure 3:**
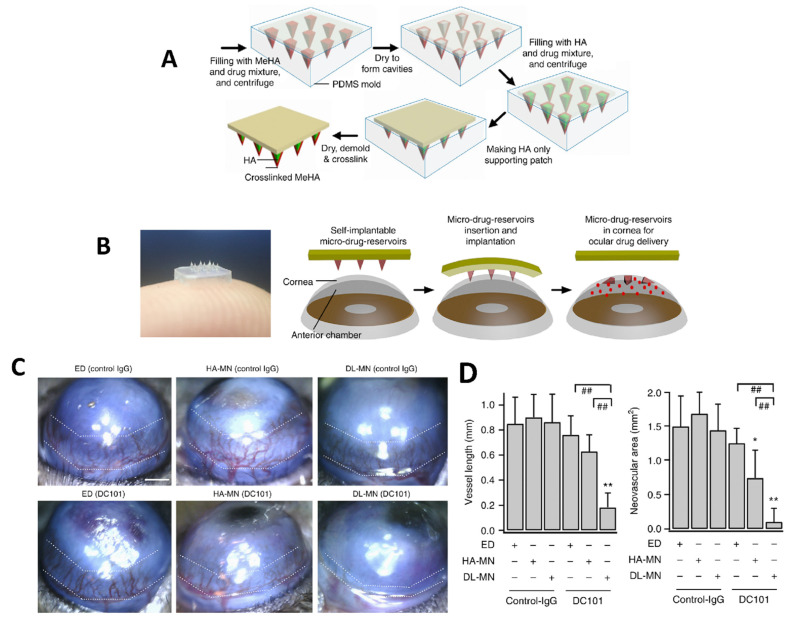
(**A**) Manufacturing process of the ocular polymeric patch equipped with MNs, (**B**) Schematic of MN-equipped eye-contact patch for ocular drug delivery, (**C**) Representative images of eyes treated with MNs. Scale bar = 500 µm, (**D**) Quantifications of corneal neovascularization (mean ± SEM; 6 samples for each group). The white dotted lines represent the extent of neovascular outgrowth from the limbus (* *p* < 0.05, ** *p* < 0.01 vs. control, ## *p* < 0.01 between indicated pairs). Reprinted from [112] with permission from Springer Nature.

Dissolving polymeric MNs are also widely used in the treatment of ocular diseases [106,107]. These systems are generally manufactured by the encapsulation of active ingredients into the polymers. They are produced using several biodegradable and biocompatible polymers that are easily insertable into the ocular tissue. They are minimally invasive systems alternative to solutions, gels, intracorneal injections, and colloidal suspensions. More recently, patches of dissolvable MNs have been used for ocular drug delivery [91]. After the MN patch has been successfully implanted into the ocular tissue, the medication inside the polymeric needle patch will begin to penetrate/release into the ocular tissue [108,109]. It should be noted that once this type of MN is applied, its removal is not possible [110]. Dissolving polymeric MNs are widely produced using polyvinyl alcohol (PVA) and polyvinyl pyrrolidone (PVP) polymers. In addition, water-soluble polysaccharides such as dextran, hydroxypropyl methylcellulose (HPMC), hydroxypropyl cellulose (HPC), sodium alginate, and hyaluronic acid are suitable polymers [111]. Than et al. developed a drug-loaded eye patch equipped with polymeric double-layered MNs (DL-MN) which were made of crosslinked methacrylated hyaluronic (MeHA) polymer and provided a controlled drug release profile (Figure 3A). The obtained self-implantable micro-drug-reservoirs system could easily penetrate into ocular tissues and allow controlled release of contained drug (Figure 3B) [112]. In the study, corneal neovascularization was utilized as the ocular disease model and the active ingredients were an anti-angiogenic monoclonal antibody (DC101) and an anti-inflammatory drug (diclofenac). The prepared MN patches were administered to the central cornea of mice for 30s and bright-field microscope images of the corneas were taken. The representative images showed that the MN patches reduced neovascular area (Figure 3C). On the other hand, mice were divided into 6 groups, then the eye-drops of non-specific control IgG or anti-VEGFR2-IgG (DC101), their individual HA-only MNs and DL-MNs were applied to the groups, respectively. As a result, DC101 containing DL-MNs exhibited the highest therapeutic effect with a 90% reduction of neovascular area (0.12 ± 0.17 mm^2^) (Figure 3D). In comparison with conventional methods, this method is less intensive, more cost-effective, and could be even used by the patient at home.

#### 4.2.2. Applications of Microneedles in Ocular Diseases

The most common use for MNs is transdermal administration, which may be used to treat both local and systemic conditions. Eye drop formulations have a high patient compliance rate; nevertheless, medications administered using this manner have difficulty in penetrating into the strong ocular barrier and reaching the ocular tissues they are intended to treat [113,114]. Intravitreal injections can bypass ocular barriers, but they are intrusive, dangerous, and must be performed by a trained specialist. MNs are well-designed systems, and they are applicable to various eye diseases. They provide an uncomplicated treatment, require just a low level of invasiveness, and are effective, all while posing a low risk of unpleasant side effects.

Uveitis is a type of inflammatory and infectious disease of the eye. Treatment of uveitis requires the use of corticosteroid, immunosuppressant, cytotoxic, biological or non-corticosteroid (e.g., anti-VEGF agents) drug medications. To treat uveitis, topical dexamethasone (DEX) administration is commonly preferred; however, the amount of the drug reaching to the back of the eye is not sufficient in topical administration [115]. On the other hand, intravitreal injection of DEX has also drawn attention. This type of administration is accompanied by undesired effects such as pain, retinal detachment, endophthalmitis, etc. To improve patient compliance, novel drug delivery systems such as MNs have been developed. In a current study, a combined dissolving MN system, that generates thermoresponsive in situ nanomicelles, has been fabricated for the effective delivery of the poorly water-soluble drug, DEX, for the treatment of uveitis. For this purpose, thermoresponsive in-situ gel-forming co-polymers were used to prepare MNs. These MNs generate nanomicelles at physiological temperature. In this study, in vitro drug permeation studies were carried out both for DEX solution and MNs. DEX solution and DEX-loaded MNs were administered to bovine scleral tissue. As a result, the scleral permeation from DEX-MNs was found to be much higher than from DEX solution [116].

**Figure 4:**
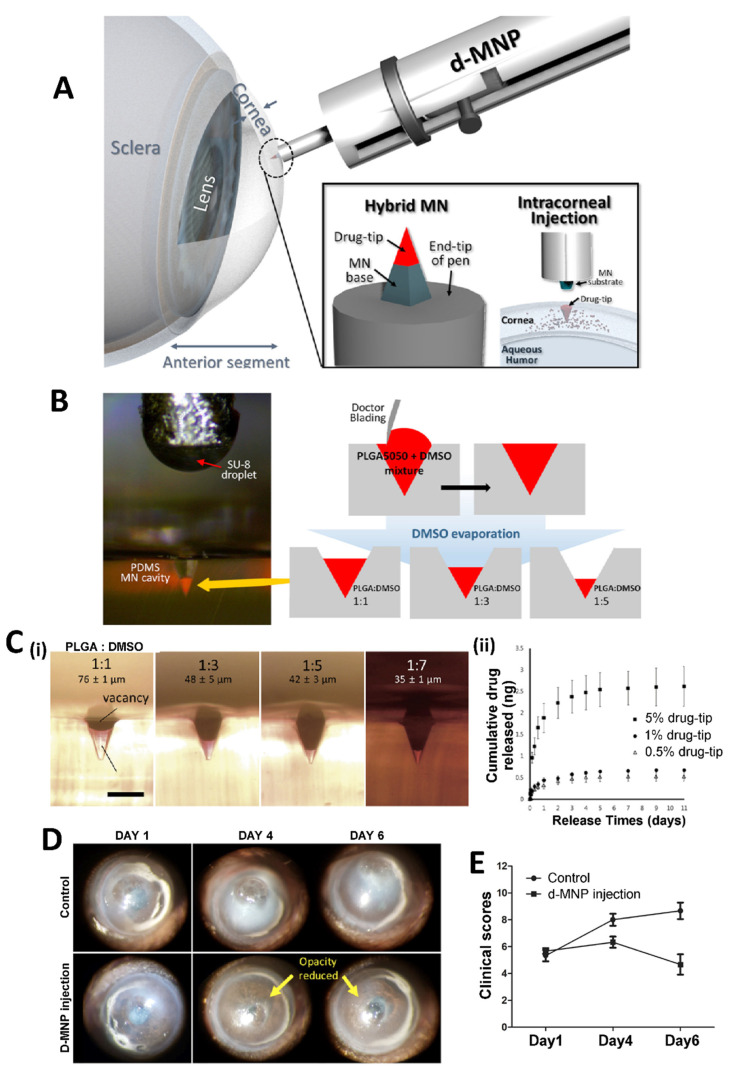
(**A**) Schematic illustration of a detachable hybrid d-MNP system and its intracorneal injection. (**B**) Dimension optimization of a drug-tip by adjusting the PLGA concentration in the drug formulation and evaporating DMSO in the molding process of the drug formulation. (**C**) Premolded PDMS mold having a drug-tip-controlled height (scale bar = 100 µm) (i), in vitro cumulative release of model drug from 5%, 1%, and 0.5% drug-tips in PBS buffer solution (ii). (**D**) The representative images of *Acanthamoeba* keratitis for 7 days in control corneas (un-treated group) and corneas treated with d-MNPs loaded with PHMB (d-MNP injection). (**E**) Clinical scores detected at each time point. Reprinted with permission from Ref. [119]. 2022, Elsevier.

MNs can also be used in the treatment of AMD, a type of posterior segment eye disease. AMD is a common eye condition, that affects older people and causes loss of vision. There are various treatment approaches for AMD and the MN-based drug delivery system is one of them. Kim et al. investigated bevacizumab (an anti-VEGF agent) coated MNs and compared the obtained results with those from topical and subconjunctival delivery of the drug. In this study, drug coated MNs demonstrated a targeted drug delivery to treat corneal neovascularization. The MNs also reduced the side effects of the drug, and they were found to be promising for the effective treatment of AMD [117]. MNs can also be combined with other technologies. Nanoparticle loaded dissolving MNs can be prepared as a hybrid system to carry proteins/peptides/biomacromolecules to the eye. For instance, the long-acting release property of polymeric poly lactic-co-glycolic acid (PLGA) nanoparticles and the delivery capability of MNs to the posterior ocular tissue have been combined to prepare a new system which exhibits an effective and sustained delivery of ranibizumab, as an optimized treatment approach for AMD [118]. There is another interesting approach to hybrid MNs in the literature (Figure 4A). For an effective delivery of drugs into cornea, a detachable and biodegradable hybrid MN system can be produced which comprises a supporting base and a drug containing a biodegradable tip to obtain a sustained release drug profile. In a study by Lee et al., MNs were manufactured using PLGA as the biodegradable polymer, polydimethylsiloxane (PDMS) as the filling molds, and dimethyl sulfoxide (DMSO) as the solvent (Figure 4B). The mentioned MN system was developed for the treatment of an infectious eye disease (*Acanthamoeba* keratitis). In the study, mouse corneas were intrastromally injected with amoebae to obtain a *Acanthamoeba* keratitis disease model. After 1 day from the inoculation, the d-MNP system was inserted into the corneal tissue for the treatment of the disease. The height of the drug-tip created in the PDMS mold demonstrated that the detachable microneedle pen (d-MNP) inserted with the drug-tip deeper than its height provided sustained release of the model drug (Figure 4C). According to Figure 4D,E, the progressing corneal opacities showed that untreated corneas exhibited marked progression of the infection. However, corneas injected with d-MNPs were less opaque than control samples (3 days after the injection, at the 4th day). The study showed the effective and sustainable use of MNs in the treatment of infectious eye diseases [119].

Retinal vascular occlusion is a retinal disorder that occurs when a blood clot blocks the retinal vein. In some cases, it causes loss of vision. The use of stainless steel MNs has been investigated to dilate the retinal vein when occlusion happened. The obtained MNs improved ocular vision and caused few complications [120]. MNs are also used for arterial cannulation to treat an embolism in the retinal artery. For this purpose, a 47-gauge stainless steel MN has been developed to fit the tiny structure of the retinal artery. The obtained MNs were applied to the patient’s eye with injection of tissue plasminogen activator (tPA) and improved visual acuity [121].

MNs could also be used for gene therapy via participating in the injection process of the gene carrier. In a study by Chadderton et al., RNAi carrying Adeno associated virus (AAV) based vector has been developed and administered by injection using a 34-gauge MN. The electroretinography results showed that the obtained system was found to be effective for gene delivery since it provided an RNAi-based suppression and replacement in an animal model of a retinal disease. Therefore, this therapeutic approach is promising for the treatment of other inherited disorders [122].

Glaucoma is a common type of posterior segment eye disease, that damages the optic nerve. MNs are one of the advantageous options in the treatment of glaucoma because they are minimally invasive systems, and they allow long-term treatment. For instance, pilocarpine-coated stainless steel MNs were administered using intrascleral and intracorneal approaches to increase the absorption rate of pilocarpine in the treatment of glaucoma. MNs were applied to rabbit eye and led to increased bioavailability in the cornea [111,123].

### 4.3. Tissue Adhesives and Their Ocular Applications

Tissue adhesives (TAs) establish applicable adhesion to tissues. TAs are clinically used for wound closure for small to life-threatening tissue damage. Compared to conventional suture or stapling applications, TAs are relatively easy to implement, have minimally invasive procedures, and enable rapid application as well as minimizing the post-operative complications [124,125]. Utilization of TAs is categorized into 3 groups: (i) hemostasis, (ii) tissue sealing by preventing leaks of different items, such as lymphatic fluids and air and (iii) local delivery of exogenous elements [126,127,128].

As the gold standard, sutures have been used for laceration repair in ophthalmology, while their application is very time-consuming and challenging and needs technical expertise. Additionally, sutured areas may cause numerous potential complications (e.g., leakage, inflammation, secondary damage, and secondary infection) [129]. TAs contribute promising advantages in ophthalmic surgery to overcome the drawback of sutures [130]. Various TAs (e.g., cyanoacrylates, collagen-based, albumin-based and glutaraldehyde-based formulations) have been developed and commercialized [124].

TAs, both biological and synthetic, have a long history of use in ophthalmology [131]. Fibrin-based TAs are used as an alternative to cyanoacrylate-based TAs to heal corneal thinning and minor perforations, possibly resulting in fewer corneal and conjunctival inflammatory reactions. Thus, fibrin-based TAs are the most prevalent TAs in recent years. In addition, they have progressively been used in various ocular operations, including pterygium surgery, forniceal reconstruction, amniotic membrane transplantation, lamellar corneal grafting, corneal flap tears and dislocations, corneal melts, and cataract [132,133]. However, their extensive usage has some limitations due to the risk factors in viral transmission and their complex preparation and application procedures [133]. One more limitation of fibrin-based TAs is their relatively short half-life in ocular tissues (<2 weeks) which restricts their use in specific situations or where there is a need for repetition [131].

One of the common anterior segment eye diseases is the dry eye which causes discomfort and impaired vision, affecting human life quality. Numerous drugs formulated in eye drops to manage dry eye are hardly soluble in an aqueous environment and are quickly eliminated from the ocular surface and so their therapeutic results are restricted [134]. To overcome these restrictions, Han et al. developed FK506-loaded thermoresponsive hydrogel-based ocular TAs using PEG- poly(propylene glycol) (PPG) copolymer functionalized with polyhedral oligomeric silsesquioxane units. According to the findings, the developed TAs showed excellent application prospects for improving long-acting ophthalmic treatments for more effective management of ocular diseases. Delivery of FK506 from bio-adhesive hydrogel showed a perfect potential application to extend drug residence time on the corneal surface, which enhances its therapeutic benefit in dry eye management [134].

**Figure 5:**
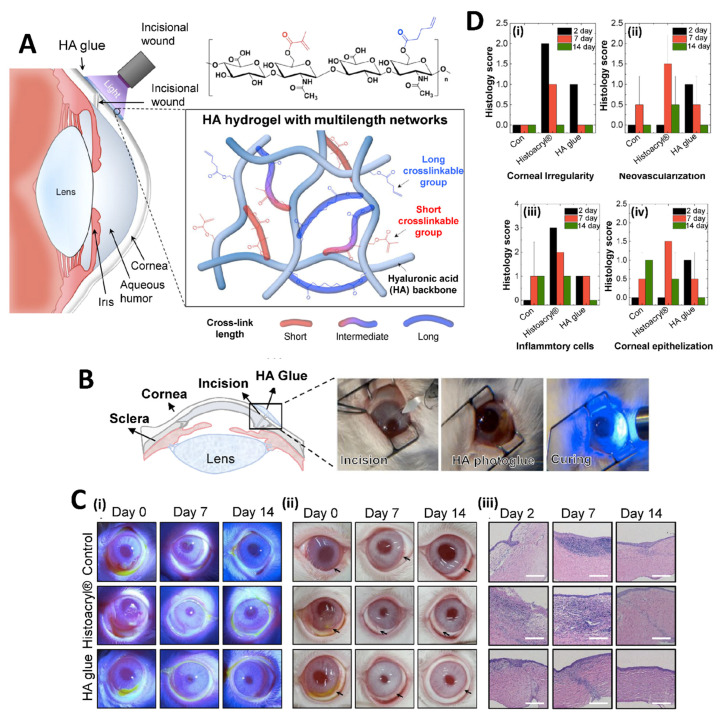
Illustration of the structure of photo-crosslinkable HA Tas and its application practice (**A**), images of in vivo experimental procedure for sealing of rabbit corneal incision using HA Tas (**B**), photographs at different time points after the incision and histological images (Scale bar: 250 μm) (**C**) and histological scores. The scores are referred to as follows: 3 (Severe), 2 (Moderate), 1 (Mild), and 0 (Negligible) (**D**). Reprinted from [129].

For the principal usage of precise corneal incisions, ReSure^®^ Sealant, developed by Ocular Therapeutix, is the only hydrogel-based TA approved by the FDA for the primary use of sealing precise corneal incisions. ReSure^®^ Sealant is a polyethylene glycol (PEG)-based TA [135]. On the other hand, light-activated ocular TAs and other new ones have been reported; however, their approval into general applications has not been determined yet. An increasing number of studies focus on this field and encourage development of candidate materials. Sani et al. engineered a highly biocompatible, transparent, and visible light-curable gelatin-based bio-adhesive (GelCORE) for corneal reconstructions. It was reported that GelCORE allows normal regenerative responses when applied to the injured corneal stromal in differential geometry and size [136]. Khalil et al. investigated the potentiality of ciprofloxacin-encapsulated micelles, including GelCORE, for eye infection prevention. GelCORE represents an encouraging system for drug elution for various corneal surface disorders [137]. Rumeysa et al. developed photo-crosslinkable GelMA/silk fibroin Tas to reconstruct ocular tissue injuries. The formulated adhesives provided strong adhesion, physical durability, and non-toxic properties. Silk fibroin composition in the formulations notably enhanced the corneal adhesion strength of the pristine GelMA Tas [138]. In another study, photo-crosslinkable hyaluronic acid (HA)-based Tas with cornea-like optical transparency was successfully synthesized to achieve firm tissue adhesion under dynamic and wet environments. HA-based Tas demonstrated an enhanced wound healing activity in the rabbit corneal incision model (Figure 5) [129].

Maeng et al. developed a blue light-curable and bioengineered mussel adhesive protein (MAP) -based TAs (named FixLight) for conjunctiva reconstruction after pterygium removal. In vivo studies of FixLight did not show any inflammatory reactions or fibrosis and was safe to achieve a complete rebuilding of conjunctiva. In addition, FixLight showed strong, rapid and wet adhesion compared to current transplanted amniotic membrane procedures that need fibrin glue and suture. Accordingly, FixLight was introduced as a clinical TA candidate in ophthalmological operations [139].

### 4.4. Other Technologies

Apart from those mentioned above, there are many technologies used in the treatment of ocular diseases. Microelectromechanical systems (MEMS), which are also known as micromachines or microsystems technology, are systems ranging in size from nano to millimeters. They contain both mechanical and electrical components. Without invasive operations, implantable medication delivery pumps may enable externally controlled variable-rate distribution. Instead of relying on erosion or diffusion to release the medicine, pressure affects the delivery [140]. Implantable infusion pumps can be classified into two groups according to their pumping mechanisms: The first one is an osmotic or fluorocarbon propellant controlled passive pump and the latter one is the electrically powered active pump. MEMs-based infusion pumps are systems that provide a controllable drug release profile, an optimized drug action selectivity, and a decreased dosing frequency [140]. Implantable medication delivery pumps on ocular processing have made considerable strides recently, promising patients more independence and a better quality of life [141]. Incorporating physical ocular sensors that provide information on pressure, flow rate, or otherwise report to the user the state of the pump or confirm delivery volumes is an exciting next step for implantable pumps; ocular sensors that are suitable for direct integration into MEMS pumps are currently in development. For the drug’s activity to have the intended therapeutic impact, advances in biological sensors are needed to allow physiological feedback to the pump [142]. Local or distant ocular sensors may be inserted in addition to the pump [143]. MEMs enabled implants are widely used for the management and treatment of chronic eye diseases such as glaucoma. In glaucoma cases, various implants with modified release properties are utilized to reduce intraocular pressure (IOP) [144]. Implant systems are also developed as glaucoma monitoring diseases. IOP monitoring sensors which are non-invasive or minimally invasive are used for this purpose [144,145].

3D-printing has an important role in the development of customized products for the treatment of ocular diseases. Using 3D-printing methods, it is possible to customize the implant formats and sizes according to the eye anatomy of the patients. The development of tailored ocular prostheses for the patients with eye loss is a long, hard, and expensive process, however. It is possible to obtain desired objects via user-controllable computer aided designs in 3D-printing [146,147]. Therefore, manufacturing a patient-specific ocular prosthesis by the means of 3D-printing is available. It is also possible to manufacture 3D-printed pupil expansion devices and obtained devices have been used in cataract surgeries for an effective pupillary dilatation. Bioprinting is also among the 3D-printing technologies. Unlike other 3D printing techniques, it allows the production of tissues and organs using biomimetic and biocompatible materials [148,149]. It is an emerging technology for the fabrication of complex-structured tissue and organs. Therefore, it is a promising technology for the replacement of tissues such as cornea, retina, and tarsal plate in cornea, external eye, and other ocular diseases [147]. In ocular diseases 3D-printed drug delivery systems are also manufactured for “personalized medicine” applications. For instance, drug-eluting implants for the treatment of glaucoma and hydrogel-based antibiotic containing patch formulations for infectious diseases of eye have been successfully developed via 3D-printing [150].

**Figure 6:**
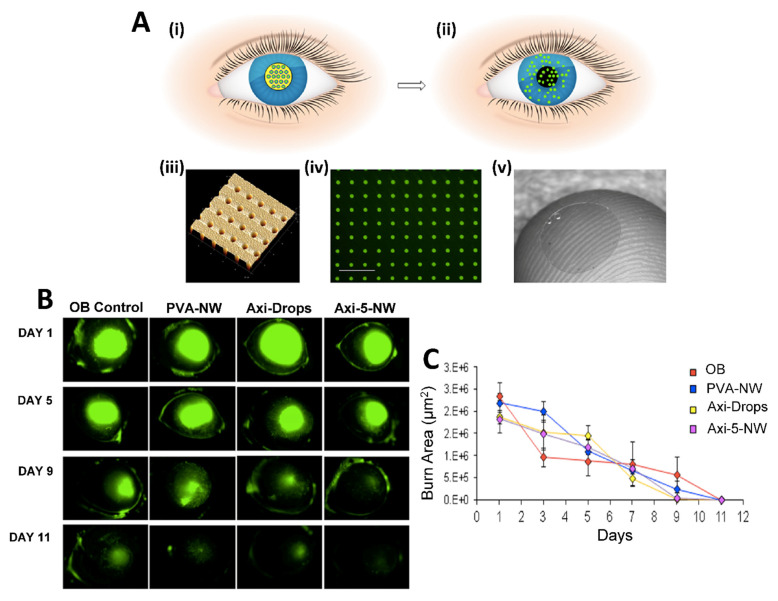
(**A**) (i) Schematic of the nanowafer being implanted on the cornea. (ii) Drug diffusion into the corneal tissue. (iii) Atomic force microscope image of a nanowafer displaying an array of 500 nm diameter nanoreservoirs. (iv) Fluorescence micrograph of a drug (doxycycline) loaded nanowafer (scale bar 5 μm). (v) Nanowafer on a fingertip. (**B**) Fluorescence images of mouse cornea to which was applied axitinib loaded nanowafers. (**C**) Graphic demonstrating corneal surface healing period. Reprinted with permission from [154] under Editors’ Choice Usage Agreement, American Chemical Society.

In situ gels are polymeric liquid formulations that transform into gels in physiological conditions. They are smart gel systems and their transformation from solution to gel form is induced by temperature, pH or other environmental conditions. The most frequently used in situ gel-forming polymers are thermosensitive polymers such as Poloxamers (Pluronics^®^). By the preparation of in situ gels, it is possible to improve solubility and bioavailability of drugs. For instance, a thermosensitive ocular gel formulation of a poorly water soluble (itraconazole) drug has been produced for effective treatment of fungal keratitis [151]. Nanoemulsion is another nano-sized system with transparent or translucent properties. It is kinetically stable based on the interaction of the oil and water phase and addition of surfactant and co-surfactant. Nanoemulsions can be integrated into thermosensitive in-situ forming gels for increased permeability and sustained release. For instance, nucleoside analog acyclovir (ACV) loaded triacetin, Transcutol^®^ P, Poloxamer 407, and Poloxamer 188 consisting of thermosensitive in situ gel nanoemulsion showed non-irritant properties in ex vivo and in vivo tests and exhibited 74.44–80.78% release efficiency for all nanoemulsion formulations against ocular Herpes simplex keratitis (HSK) infection [152]. Nanowafers are another type of material with small transparent rectangular or circular disc-like shape and drug loading ability which could be used as an effective treatment method for ocular diseases [58,153]. Nanowafers release the drug in a slow manner (Figure 6A); thus, they provide an enhanced drug-residence time and bioavailability on ocular surface and tissues. Nanowafers can be applied with a high patient compliance as they allow self-medication like contact lenses. It is possible to produce nanowafers using mucoadhesive hydrogel-forming polymers such as carboxymethyl cellulose (CMC). Coursey et al. developed dexamethasone-loaded long acting nanowafers for an effective treatment of dry eye disease. In a study by Yuan et al., axitinib and doxycycline loaded nanowafers were manufactured using different polymers. The axitinib nanowafers released the drug over a prolonged period as is concluded from fluorescence microscope images of the mouse cornea (Figure 6B). Axitinib loaded nanowafers were found to be effective for the treatment of corneal neovascularization induced by burn in animal models [154,155] (Figure 6C). In conclusion, the nanowafer drug delivery system increased the therapeutic efficacy of drugs since it provided an enhanced drug residence time on the ocular surface, released the drugs in a controlled manner and improved their bioavailability. The nanowafer system also inhibited neovascularization and reduced the suppression of proangiogenic and proinflammatory factors in the ocular burn induced animal model.

## 5. Preclinical and Clinical Findings

As mentioned before, the current approaches used for the treatment of most of ocular disease are invasive and bothersome for most patients. This has led to a wide range of research to find new methods with less invasiveness and probable better performance. Among them are the application of contact lenses, microneedles, in situ gels, etc., in combination with nanomaterials, some of which have reached the clinical evaluation and even commercial level. For instance, different types of drug laden contact lenses have been evaluated to detect their effectiveness in comparison with the commercial eye-drops. Utilizing Vasurfilcon A contact lens soaked in timolol and brimonidine, 30 min per day for 2 weeks, led to improvements in the signs of glaucoma in comparison to using eye-drops. Controlled release of gentamicin from the soaked contact lenses for two weeks also showed better performance against the application of commercial gentamicin eye-drops for the treatment of external ocular infections [156,157]. Functionalizing contact lenses with different types of nanomaterials could fabricate formulations with interesting features such as controlled release capability, and diagnostic ability, as well as reducing the frequency of dosing, increasing the stability and shelf-life of the therapeutic agent, greater convenience, and enhancing the performance of treatment [158,159,160,161]. In these cases, there are some patents on the application of contact lenses for drug delivery applications that are summarized in Table 2.

To be commercialized these products should pass both preclinical and clinical levels. Indeed, most of them have passed the preclinical tests and now are in their clinical trials progression. For instance, the effectiveness of ceria nanoparticles embedded in contact lenses was tested and confirmed in a preclinical test. It could scavenge the excessive reactive oxygen species produced in eyes, and so could be effective in preventing different types of ocular disease [162]. CLs containing dexamethasone have been tested at Massachusetts Eye and Ear Infirmary for the treatment of cystoid macular edema at clinical level [61]. A group at Harvard University is working on latanoprost eluting contact lenses to examine their tolerability, safety, comfortability, and feasibility for controlling IOP [163].

Application of smart lenses with high accuracy in detecting the symptoms of disease is in high interest in recent years. These are new technologies which are mostly in preclinical level tests and are used for real-time and continuous monitoring of disease symptoms and controlled release of drugs for the disease treatment [164].

Besides the CLs, various new technologies were also evaluated by the preclinical and clinical studies. For example, the intraocular drug delivery was mediated by a self-plugging MN which is composed of polylactic acid (PLA) coated by methacrylated hyaluronic acid hydrogel, to provide the self-plugging ability, and poly lactic glycolic acid (PLGA) as the drug carrier. The fabricated MNs provide capability of drug delivery to different locations inside the eye via controlling its shape and length. Moreover, preclinical investigations confirmed non-invasiveness of this MN due to the maintenance of integrity in the surrounding scleral tissue [165]. Microneedles have the capability of drug delivery into both anterior and posterior site of the eye. Important points that should be considered for the preclinical and clinical applications of MNs include: (a) selecting appropriate animal models for in vivo evaluation with high similarity to humans; (b) assessment of biocompatibility and cytotoxicity of fabricated MNs in both animal models and humans; (c) introducing optimal guidelines for both preclinical and clinical tests with high efficiency and reusability, and (d) complete understanding of the biological and pharmaceutical effects (both pharmacokinetic and pharmacodynamic effects) of the MNs on both animals and humans [166].

In situ gels are among other current technologies used for the treatment of ocular disease. In this context, the application of thermo-responsive poly(α-carboxylate-*co*-α-benzylcarboxylate-ε-caprolactone)-*block*-poly(ethylene glycol)-*block*-poly(α-carboxylate-*co*-α-benzylcarboxylate-ε-caprolactone) (PCBCL-*b*-PEG-*b*-PCBCL) gel, as carrier for the delivery of cyclosporine A to the eyes, was evaluated and compared with Restasis^®^ eye drop on rabbits. The results of this study showed enhanced performance using the fabricated hydrogel, with better tolerance, lower irritation and sustained release of the drug [167]. Poly (D, L-lactide-co-glycolide) nanoparticles contained pioglitazone, which was dispersed in an in situ poloxamer 407 and Hydroxypropylmethylcellulose (HPMC) K4M gel. In comparison to the marketed formulation, the fabricated hydrogel showed a non-irradiation effect that could induce tear production and stabilize the tear film for a long period [168]. The effectiveness of these gels was confirmed in different preclinical tests for the treatment of different diseases such as conjunctivitis, dry eye, cataracts, uveitis, glaucoma, and infections; however, the number of clinical studies is restricted in this case and more research are needed in this field [169]. Some of the other kinds of advanced technologies which were for preclinical or clinical detection and treatment of different types of ocular disease are summarized in Table 3.

## 6. Conclusions, Limitations, and Future Perspectives

Advanced ocular drug delivery has been one of the most interesting fields of research in the last 30 years, with two important goals: improving the bioavailability of drugs inside the eyes and so overcoming the anatomical and physiological barriers, and enhancing their performance, especially in the case of disease related to the posterior side of the eye. Accordingly, some new techniques are introduced to overcome the limitations of current methods. Nanotechnology is one of these techniques, giving the ability to deliver drugs to different parts of the eye. To this end, different types of nanomaterials are being introduced to improve the bioavailability of therapeutic components and their diffusion to their targeted site in eyes along with reducing the side effects of the therapeutic components. Recently, the combination of nanotechnology with other techniques such as contact lens and microneedles has led to new treatment methods with better performance, some of which are summarized in the above sections. Despite the progress made for the fabrication of new methods for the treatment of ocular disease, there are still some limitations about their usage, especially for the clinical applications. For instance, contact lens have limitations in sterilization, packaging and shelf life, insertion and removal of lens, as well as high risk of bacterial contamination. In addition, utilizing preservatives to limit the probable contamination could affect the amounts of drug loading and kinetics of release. Moreover, lack of compatibility between the ocular surface and the lens could lead to ocular sensations that may affect the visual acuity. Indeed, possible toxicity effects and contamination are the typical challenges related to all the previously mentioned methods. The other important point is the probable effects of these techniques on the physiological features of the eye (such as decreasing the pH, enhancing the hypoxic condition, mechanical pressure, and osmolarity) which might lead to other disorders. One of the important points to keep in mind is the material selection, which could directly affect features of these drug delivery techniques. Accordingly, considering the basic physical and chemical properties of materials used for fabrication (such as good oxygen permeability, high biocompatibility and high drug loading capacity) could improve their performance. The other important point is determining the type of drug according to type of disease, which also could determine the type of material used. In this context, new techniques such as biomedical microelectromechanical systems (bioMEMS) or lab-on-a-chip and smart materials could be beneficial for real-time detection of the performance via reducing the number of preclinical and clinical tests. The other important point is reducing the public fear about the use of nanomaterials by increasing public awareness of their benefits as well as presenting accurate test results.

## Figures and Tables

**Table 1:** Ocular studies with drug/nanoparticle incorporated in contact lenses.

Nanoparticle Type	Model Drug/Active Ingredient	Lens Material	Disease	Main Results	Ref.
Micelle	Cyclosporine	HEMA, EGDMA, Irgacure contact lenses	Dry-eye syndrome	The therapeutic effect of micelle-embedded CLs on dry eye syndrome was confirmed by corneal fluorescein staining, Schirmer tear test, and MMP9 fluorescein analysis in DED model rabbits.	[62]
Zwitterionic nanogels based on poly(sulfobetaine methacrylate) (PSBMA)	Levofloxacin	Soft contact lenses	-	Release from CLs occurred during 10 days with critical lens properties within the range of recommended values.	[63]
Liposome	Levofloxacin	HEMA, PVP, EGDMA, 2,20-azobis(2-methylpropionitrile) (AIBN) contact lenses	Ocular infections	Friction did not significantly affect drug release from liposome-coated lenses and increasing the temperature resulted in an increase in drug diffusion rate.	[64]
Micelle	Orfloxacin and puerarin	Hydrogel contact lens	Ocular infections	The results showed that the polymer micelle puerarin in the hydrogel could remain stable in hydrogels and slow the release rate of orfloxacin.	[65]
Micelle	Dexamethasone acetate	Poly (2-hydroxyethyl methacrylate–methacrylic acid–ethylene glycol dimethacrylate) hydrogel contact lenses	Ocular infections	In vitro release of up to 30 days was observed from hydrogels prepared with micelles containing dexamethasone acetate, a hydrophobic ophthalmic drug. It may be suitable as an extended-release soft CL material.	[66]
Microemulsion	Ofloxacin	HEMA, DMA, EGDMA, Siloxane, Irgacure contact lenses	Conjunctivitis	Compared with traditional ofloxacin soaking method, the ofloxacin-microemulsion soaking solution showed a twofold increase in drug loading and sustained release up to 72–120 h. In addition, an in vivo study in rabbits shows that the developed formulation showed an equivalent curative effect compared to the commonly used high-dose eye drop treatment.	[67]
Solid lipid nanoparticle	Latanoprost	Siloxane, NVP, DMA, EGDMA, Irgacure, and HEMA	Ocular hypertension	Pegylated lenses showed a higher drug concentration up to 96 h at all time points than eye drop solution in in vivo studies.	[68]
Propoxylated glyceryl triacrylate nanoparticle	Timolol maleate	Silicone hydrogel (Acuvue Oasys) contact lenses	Glaucoma	The nanoparticles release the drug for a long time due to the slow hydrolysis of the ester bond. Lenses loaded with 5% nanoparticles with a 1:1 timolol:PGT ratio had minimal effect on critical properties and released therapeutic drug doses.It has been found to be effective and safe in the treatment of glaucoma in preliminary in vivo animal studies.	[69]
Gold nanoparticle	Timolol maleate	Hydroxyethyl methyl acrylate (HEMA), dimethyl acrylamide (DMA), ethylene glycol dimethyl acrylate (EGDMA), N-vinyl pyrrolidone (NVP), 3-[Tris(trimethylsiloxy)silyl]propyl methacrylate (siloxane) and photoinitiator (Irgacure)	Glaucoma	With the developed formulation, the concentration of timolol in the tear fluid is higher at all time points than with the conventional wetting method.	[70]
Eudragit nanoparticle	Levobunolol	Hilafilcon B (Iconnect), Omafilcon A (Slip-on), Polymacon (Optima 38), Etafilcon A (Acuvue Moist), Hilafilcon B (Softlens) hydrogel contact lenses	Glaucoma	In vitro release studies showed an initial immediate release followed by a sustained therapeutic release for up to 12 days. The drug is a controlled release from pH-sensitive Eudragit S100 nanoparticles.	[71]
Silica nanoparticle	Brimonidine tartrate	Siloxane, DMA, NVP, EGDMA	Glaucoma	Silica nanoparticles showed higher drug concentration at all time points up to 96 h compared to eye drops, resulting in prolonged drug release.	[72]
Micelle	Timolol and latanoprost	HEMA, EGDMA contact lenses	Glaucoma	Sustained drug release in lacrimal fluid over 96 h for latanoprost and >120 h for timolol in vivo. In this case, it is thought that there may be an increase in the bioavailability of the drugs.	[73]
Microemulsion	Timolol maleate	HEMA, Irgacure, DMA, EGDMA, siloxane contact lenses	Glaucoma	It has been observed in in vivo tear experiments that microemulsions prevent immediate drug release compared to direct drug immersion and are effective for 96 h.	[74]
Microemulsion	Travoprost	HEMA, DMA, EGDMA, Siloxane, Irgacure contact lenses	Glaucoma	The use of the microemulsion system doubled the travoprost loading capacity compared to travoprost solution and extended the drug release to 48–120 h. It can be used for other BCS class II drugs with the improvement of the release profile.	[75]
Microemulsion	Bimatoprost	HEMA, DMA, EGDMA, Siloxane, Irgacure contact lenses	Glaucoma	When using bimatoprost-microemulsion soaking solution compared to bimatoprost-soaking solution, drug loading increased by two fold, and drug release increased up to 48–96 h. In addition, histopathological studies of corneas showed normal squamous epithelial cells.	[76]
Liposome	Carboxyfluorescein	Hioxifilcon B contact lenses	-	The risk of separating the prepared multilayered liposomes from the CL surfaces is shallow with covalent or biotin/avidin affinity. They can provide diffusion-controlled release of antibiotics to treat ocular bacterial infection.	[77]

**Table 2:** Patents related to the therapeutic application of contact lens. Reprinted with permission from Ref. [158]. 2023, Elsevier.

No.	Patent No.	Year	Title	Scope
1	US8349352B2	2007	Therapeutic contact lenses with anti-fungal delivery	Molecular imprinted approach to fabricate drug-eluting contact lens. Use of recognitive polymeric hydrogel by using bio-template for antimicrobial or fungal ocular drug delivery
2	US8388995B1	2009	Controlled and extended delivery of hyaluronic acid and comfort molecules by a contact lens platform	The patent covers molecularly imprinted contact lenses for extended release of ketotifen fumarate, fluconazole, diclofenac sodium, hyaluronic acid, and hydroxypropyl methylcellulose
3	US8273366B2	2004	Ophthalmic drug delivery system	The patent covers the loading of nanoparticle dispersion into contact lens for ocular drug delivery
4	US8623400B2	2011	Drug-carrying contact lens and method for fabricating the same	The patent covers the biocompatible hybrid nanocarriers loaded contact lens and method for the fabrication of the same. The invention also includes the heat and light-sensitive drug molecules drug encapsulation in hybrid nanocarriers incorporation into contact lens for ocular drug delivery
5	WO2011053633A1	2011	Fast-response photochromic nanostructured contact lenses	Incorporation of photochromic agents in a contact lens to protect eyes from harmful light
6	WO2010022056A1	2008	Microbial cellulose contact lens	Contact lens fabrication from *Gluconacetobacter xylinus* cellulose material and the use of the same for corrective and non-corrective vision
7	US20140377327A1	2014	Extended-release drug-delivery contact lenses and methods of making them	Fabrication of prolonged drug-releasing contact lens by electrospinning technology with improved oxygen permeability
8	WO2009094466A3	2008	Contact lenses for extended-release of bioactive agents containing diffusion attenuators	Silicone hydrogel contact lens comprising diffusion barrier such as vitamin E to extend the drug release for a more extended period
9	US8414926B1	2007	Nanoparticles with covalently bound surfactant for drug delivery	The patent covers the encapsulation of surfactant-free nanoparticles in a contact lens for ocular drug delivery in human and non-human subjects by covalent bonding to the polymer moiety
10	EP2978453A4	2014	Drug delivery from contact lenses with a fluidic module	Use of fluidic module in a contact lens to prevent drug release during storage conditions and to release the drug in response to the pressure of the eyelid
11	US8469934B2	2011	Pulsatile *peri*-corneal drug delivery device	The patent covers the contact lens, which releases the drug in a pulsatile manner for an extended period. Separate and distinct units are distributed in the annular reservoir and dispose of the drug as multiple different discrete units
12	WO2016171529A1	2016	Smart contact lenses and smart glasses	The patent cover preparation of a theranostic contact lens that contains a sensor for real time monitoring of disease markers and a drug reservoir for treatment
13	US9259350B2	2017	Ophthalmic devices for delivery of beneficial agents	Utilizing contact lenes containing phosphorylcholine groups releasing beneficial polyionic or guanidinium-containing agents
14	US20150305929A1	2017	Magnetic contact lenses and methods of treatment and diagnosis using the same	Application of magnetic contact lenses for magnetic diagnosis and therapy
15	US10830776B2	2020	Functionalized eyewear device for detecting biomarker in tears	Detection of specific biomarker of disease using contact lenses functionalized by aptamer molecules

**Table 3:** Some of the pre-clinical and clinical research used for the diagnosis and treatment of ocular disease.

Type of Device	Disease	Materials Used	Effects	Ref.
Contact lens	Diabetic retinopathy	Far red/NIR light emitting diode (LED), circuit chip, wireless power, communication systems on a PET film	Exhibiting the photobiomodulation effect on diabetic retinopathy	[170]
	Glaucoma	Biocompatible nano-porous material-anodic aluminum oxide (AAO) thin film	- Detection of glaucoma biomarkers - Measuring the intraocular pressure (IOP) - Extending the drug delivery in situ	[171]
	Corneal infection	Vancomycin, phenylboronic acid monomers	- Demonstrating excellent bactericidal and anti-inflammatory effects - Enhancing the bioavailability of drug on ocular surface	[172]
	Glaucoma	Nanoporous microemulsion, timolol, PNIPAM, hydroxyethylmethacrylate, ethylene glycol dimethylacrylate, 3-[*tris*(trimethylsiloxy)silyl] propyl methacrylate	- Decreasing the IOP after 2 h - Continuous therapeutic effects - Improving the bioavailability of drug	[173]
Microneedle	Age-related macular degeneration	Tower Microneedle, anti-vascular endothelial growth factor antibodies	- Drug delivery into the eye’s posterior segment - High anti-angiogenesis - Reducing the tissue damage and bleb formation	[174]
	Retinal diseases	Triamcinolone acetonide loaded MNs	Promoting transscleral penetration of drug	[175]
	Fungal keratitis	Microneedle ocular patch, liposomes contained Amphotericin B	- Significantly reducing the amounts of *Candida albicans* - Reducing the invasiveness of drug delivery	[176]
In situ gel	Glaucoma	Carbopol^®^, hydroxyl propyl methyl cellulose (HPMC), dorzolamide	- Improving the bioavailability of drug through the mucoadhesive compound - Reducing the frequency of administration - Decreasing the systemic side effects	[177]
	Fungal keratitis and endophthalmitis	Fluconazole (FL), niosomal vesicle, cationic Eudragit RS100 and Eudragit RL100, chitosan	- Enhancing drug flux - Increasing the growth inhibition - Sustaining drug release - Boosting corneal permeation - Improving the antifungal activity - Prolonging the action	[178]
	Glaucoma	Brinzolamide, poloxamer 188, Poloxamer 407, Tween 80, Triacetin, Transcutol^®^ P	- High tolerated formulation - Reducing the IOP - Enhancing the bioavailability of the drugs	[179]

## Data Availability

No new data were created or analyzed in this study. Data sharing is not applicable to this article.
